# The impact of empiric antimicrobial therapy with a β-lactam and fluoroquinolone on mortality for patients hospitalized with severe pneumonia

**DOI:** 10.1186/cc3934

**Published:** 2005-12-06

**Authors:** Eric M Mortensen, Marcos I Restrepo, Antonio Anzueto, Jacqueline Pugh

**Affiliations:** 1 Investigator, VERDICT Research Center, Audie L Murphy VA Hospital, 7400 Merton Minter Boulevard (11C6), San Antonio, TX 78229, USA; 2 Assistant Professor of Medicine, Division of General Medicine, The University of Texas Health Science Center at San Antonio, 7703 Floyd Curl Drive, San Antonio, TX 78229, USA; 3 Investigator, VERDICT Research Center, Audie L Murphy VA Hospital, 7400 Merton Minter Boulevard (11C6), San Antonio, TX 78229; 4 Assistant Professor of Medicine, Division of Pulmonary and Critical Care Medicine, The University of Texas Health Science Center at San Antonio, 7703 Floyd Curl Drive, San Antonio, TX 78229, USA; 5 Professor of Medicine, Division of Pulmonary and Critical Care Medicine, The University of Texas Health Science Center at San Antonio, 7703 Floyd Curl Drive, San Antonio, TX 78229, USA; 6 Director, VERDICT Research Center, Audie L Murphy VA Hospital, 7400 Merton Minter Boulevard (11C6) San Antonio, TX 78229, USA; 7 Professor of Medicine, Division of General Medicine, The University of Texas Health Science Center at San Antonio, 7703 Floyd Curl Drive, San Antonio, TX 78229, USA

## Abstract

**Introduction:**

National clinical practice guidelines have recommended specific empiric antimicrobial regimes for patients with severe community-acquired pneumonia. However, evidence confirming improved mortality with many of these regimes is lacking. Our aim was to determine the association between the empiric use of a β-lactam with fluoroquinolone, compared with other recommended antimicrobial therapies, and mortality in patients hospitalized with severe community-acquired pneumonia.

**Methods:**

A retrospective observational study was conducted at two tertiary teaching hospitals. Eligible subjects were admitted with a diagnosis of community-acquired pneumonia and had a chest X-ray and a discharge ICD-9 diagnosis consistent with this. Subjects were excluded if they received 'comfort measures only' during the admission, had been transferred from another acute care hospital, did not meet criteria for severe pneumonia, or were treated with non-guideline-concordant antibiotics. A multivariable logistic regression model was used to assess the association between 30-day mortality and the use of a β-lactam antibiotic with a fluoroquinolone compared with other guideline-concordant therapies, after adjustment for potential confounders including a propensity score.

**Results:**

Data were abstracted on 172 subjects at the two hospitals. The mean age was 63.5 years (SD 15.0). The population was 88% male; 91% were admitted through the emergency department and 62% were admitted to the intensive care unit within the first 24 hours after admission. Mortality was 19.8% at 30 days. After adjustment for potential confounders the use of a β-lactam with a fluoroquinolone (odds ratio 2.71, 95% confidence interval 1.2 to 6.1) was associated with increased mortality.

**Conclusion:**

The use of initial empiric antimicrobial therapy with a β-lactam and a fluoroquinolone was associated with increased short-term mortality for patients with severe pneumonia in comparison with other guideline-concordant antimicrobial regimes. Further research is needed to determine the range of appropriate empiric antimicrobial therapies for patients with severe community-acquired pneumonia.

## Introduction

Community-acquired pneumonia is the seventh leading cause of death in the USA and is the leading infectious cause of death [1]. Although mortality dropped precipitously with the advent of antimicrobial therapy, since 1950 mortality has gradually increased [2].

Because of this substantial mortality, numerous societies, including the American Thoracic Society, the Infectious Diseases Society of America, and the British Thoracic Society, have published clinical practice guidelines for community-acquired pneumonia [3-9]. Although some of the content of these clinical practice guidelines is evidence-based, limited evidence is available to support many of the recommendations regarding antimicrobial therapy for patients with community-acquired pneumonia. Previous studies have suggested that the empiric use of β-lactams alone is associated with increased mortality, and that the use of macrolides for patients with community-acquired pneumonia is associated with improved outcomes [10-15]. However, few published studies have examined the combination of a β-lactam plus fluoroquinolone for patients hospitalized with severe community-acquired pneumonia, and all have had limited ability to assess the impact of this therapy [10,13,16].

The aim of this study was to assess whether the empiric use of a β-lactam with a fluoroquinolone, compared with other guideline-concordant antimicrobial therapies, has similar 30-day mortality for patients hospitalized with severe community-acquired pneumonia.

## Methods

This is a retrospective cohort study of patients hospitalized with community-acquired pneumonia at two academic tertiary care hospitals in San Antonio, TX. Both hospitals are teaching affiliates of the University of Texas Health Science Center at San Antonio. The Institutional Review Board of the University of Texas Health Science Center at San Antonio approved the research protocol with exempt status.

### Study sites/inclusion and exclusion criteria

We identified all patients admitted to the study hospitals between 1 January 1999 and 1 December 2002 with a primary discharge diagnosis of pneumonia (ICD-9 codes 480.0 to 483.99 or 485 to 487.0) or secondary discharge diagnosis of pneumonia with a primary diagnosis of respiratory failure (518.81) or sepsis (038.xx). Subjects were included if they fulfilled the following criteria: first, they were greater than 18 years of age; second, they had an admission diagnosis of community-acquired pneumonia; third, they had a radiographically confirmed infiltrate or other finding consistent with community-acquired pneumonia on chest X-ray or computerized tomography obtained within 24 hours of admission; and fourth, they met criteria for severe community-acquired pneumonia either by being in pneumonia severity index class V, meeting American Thoracic Society criteria for severe pneumonia, or being hospitalized in the intensive care unit in the first 24 hours after presentation [6,17].

Exclusion criteria included the following: first, discharge from an acute care facility within 14 days of admission; second, transfer after being admitted to another acute care hospital; third, receiving 'comfort measures only' during the admission; and fourth, receiving a non-guideline-concordant antibiotic within the first 48 hours of admission. If a subject was admitted more than once during the study period, only the first hospitalization was abstracted.

### Data abstraction

Chart review data included demographics, comorbid conditions, physical examination findings, laboratory data, and chest radiograph reports. In addition, data on important processes of care measures for patients hospitalized with community-acquired pneumonia were also abstracted: first dose of antibiotics within four hours of admission, collection of blood cultures before antibiotic administration and in the first 24 hours, and measurement of oxygen saturation within 24 hours of presentation [18].

Mortality was assessed with information from the Texas Department of Health and the Department of Veteran Affairs clinical database. Mortality status was assessed up to the end of December 2002.

### Antimicrobial therapy

We obtained information on all antimicrobial therapies given within the first 48 hours of admission. Antimicrobial regimes considered guideline-concordant included, first, β-lactam with a macrolide or anti-pneumococcal fluoroquinolone, and second, anti-pneumococcal fluoroquinolone with clindamycin, vancomycin, or an aminoglycoside (for patients allergic to penicillin) [6,7]. Antibiotics classified as β-lactams included cefuroxime, ceftriaxone, cefotaxime, cefepime, ampicillin-sulbactam, ampicillin (high dose), piperacillin-tazobactam, imipenem-cilastatin, and meropenem. Antibiotics classified as anti-pneumococcal fluoroquinolones included levofloxacin, gatifloxacin, and moxifloxacin, and antibiotics classified as macrolides included erythromycin, clarithromycin, and azithromycin. For a patient to be classified as having received a β‑lactam plus macrolide, or a β-lactam plus fluoroquinolone, they would have had to receive only those two antibiotics. Patients receiving more than two antibiotics, and who received at minimum a combination that was considered guideline concordant, were classified as having received other guideline-concordant regimes.

### Risk adjustment

The pneumonia severity index was used to assess the severity of illness at presentation [17]. The pneumonia severity index is a validated prediction rule for 30-day mortality in patients with community-acquired pneumonia. This rule is based on three demographic characteristics, five comorbid illnesses, five physical examination findings, and seven laboratory and radiographic findings from the time of presentation. Patients are classified into five risk classes with 30-day mortality ranging from 0.1% for class I to 27% for class V for patients enrolled in the original Patient Outcomes Research Team cohort study [17].

### Outcome

We used 30-day mortality as the outcome for this study. Previous research has demonstrated that 30-day mortality is due primarily to the community-acquired pneumonia rather than other co-existing co-morbid conditions [19,20]. Therefore by using 30-day mortality as our outcome we are able to examine the effect of different antimicrobial combinations on primarily pneumonia-related mortality.

### Statistical analyses

Univariate statistics were used to test the association of sociodemographic and clinical characteristics with all-cause 30-day mortality. Categorical variables were analyzed with the χ^2 ^test and continuous variables were analyzed with Student's *t *test.

A propensity score technique was used to balance covariates associated with antimicrobial therapy between groups [21-23]. The propensity score was derived from a logistic regression model. A dichotomous indicator variable indexing whether a patient received a β-lactam and fluoroquinolone was our predictor variable. To determine which covariates to include in the model we examined the univariate associations of β-lactam plus fluoroquinolone use with demographic and clinical characteristics, and included those variables that were statistically significant. In addition we included variables that we thought *a priori *would be associated with the use of different antimicrobial combinations. The covariates used in the propensity score model were pneumonia severity index, use of mechanical ventilation, admission through the emergency department, initial antibiotics within four hours, and admission to the intensive care unit within 24 hours of admission.

We used logistic regression to assess the impact of empiric antimicrobial therapy with a β-lactam plus fluoroquinolone on 30-day mortality. Covariates included in the model were the use of a β-lactam with fluoroquinolone and an ordered categorical variable based on quartile stratification on the propensity score. Model fit was assessed with the Hosmer–Lemeshow goodness-of-fit test [24]. Interactions were assessed with cross-product terms. No interactions were statistically significant, so none of the interaction terms were left in the final model.

We used a Cox proportional hazard model to estimate, and graph, the baseline survivor functions after adjusting for the propensity score.

All analyses were performed with STATA version 8 (Stata Corporation, College Station, TX, USA).

## Results

Data were abstracted on 172 patients at the two hospitals (Table 1). The mean age was 63.5 years (SD 15). The population was 88% male; 91% were admitted through the emergency department and 62% were admitted to the intensive care unit within the first 24 hours after admission. Mortality was 19.8% at 30 days. For community-acquired pneumonia-related processes of care, 33% received the initial dose of antibiotics within four hours of presentation and a further 58% received the initial antibiotic dose within eight hours, 81% of patients had blood cultures obtained within 24 hours and before the initial dose of antibiotics, and oxygenation was assessed at presentation in 87%.

The most common empiric antibiotic combinations used in this sample were ceftriaxone and azithromycin in 26%, piperacillin-tazobactam and levofloxacin in 12%, piperacillin-tazobactam and azithromycin in 8%, cefotaxime and azithromycin in 7%, ceftriaxone and levofloxacin in 7%, piperacillin-tazobactam and gatifloxacin in 5%, and ceftriaxone and gatifloxacin in 3.5%.

For subjects who received a β-lactam with fluoroquinolone, 30-day mortality was 30%, (*n *= 15 of 50), which was significantly higher than for patients receiving any other guideline concordant antimicrobial combination (*p *= 0.03). For patients who received a β-lactam with macrolides, 30-day mortality was 17.2% (15 of 87) and for other guideline-concordant antibiotic regimes mortality was 11.4% (4 of 35). When stratified by pneumonia severity index risk class, 30-day mortality was 30% (4 of 13) for patients who received a β-lactam with a fluoroquinolone, compared with 7.4% (2 of 27) for other antibiotic regimes in pneumonia severity index classes I to III, 29% (4 of 14) compared with 12% (4 of 34) in class IV, and 30% (7 of 23) compared with 21% (13 of 61) in class V. Table 2 shows the pneumonia severity index, components of the pneumonia severity index, and processes of care by β-lactam with a fluoroquinolone in comparison with other antimicrobial combinations.

**Figure 1:**
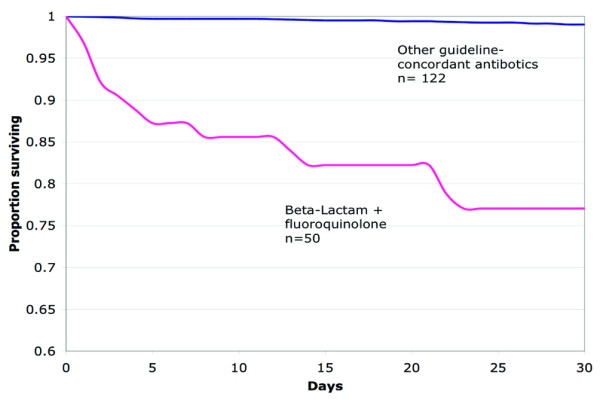
Proportion of surviving patients hospitalized with severe community-acquired pneumonia by antibiotics received (*p *= 0.004).

Figure 1 demonstrates the association of the use of a β-lactam plus a fluoroquinolone, compared with other guideline-concordant therapies, with 30-day mortality. This survival graph demonstrates that there was a significant difference (*p *= 0.004) in 30-day mortality between patients who received β‑lactams plus fluoroquinolones and patients who received other guideline-concordant antimicrobial therapies.

In this cohort, 41 patients had organisms identified from blood cultures, sputum cultures, or from *Legionella *studies including sputum direct fluorescence antibody or urinary antigen studies (Table 3). The most common organisms identified were *Streptococcus pneumoniae *in 15 isolates and *Staphylococcus aureus *in 10. As regards resistance rates of *Streptococcus pneumoniae *in our sample, 3 of 15 isolates were resistant to penicillin and 2 of 15 were resistant to fluoroquinolones.

In the multivariable analysis, after adjustment for potential confounders with the propensity score, the use of a β-lactam with a fluoroquinolone (odds ratio 2.71, 95% confidence interval 1.2 to 6.1) was significantly associated with increased 30-day mortality. Table 4 shows the results of the multivariable regression model.

## Discussion

Community-acquired pneumonia continues to be an acute medical problem, with substantial mortality and morbidity. Although our study supports many of the antimicrobial regimes suggested by the Infectious Diseases Society of America and American Thoracic Society guidelines, our study calls into question the empiric use of the antimicrobial combination of a β-lactam plus a fluoroquinolone for patients hospitalized with severe community-acquired pneumonia.

Our results strengthen the previous body of research addressing what antimicrobial therapies are appropriate for patients with severe community-acquired pneumonia. Several previous studies have found that the use of a β-lactam plus a macrolide is associated with significantly lower mortality [10-13,25]. Several studies have demonstrated that monotherapy with β‑lactam is associated with worse outcomes, including increased mortality and increased length of stay [11-13,26,27]. Several other studies have demonstrated that the use of empiric antimicrobial therapy that is concordant with national guidelines is associated with decreased mortality [12,28,29]. However, few studies have examined the combination of β-lactam antimicrobials with fluoroquinolones in comparison with other guideline-concordant strategies [10,13,16], and these studies found no significant difference between the use of a β-lactams with fluoroquinolones and other combinations. However, these studies had significant limitations including a lack of multivariate analysis and too few subjects to be able to examine this antimicrobial combination.

It is unclear why the combination of a β-lactam with a fluoroquinolone should result in significantly higher mortality than other antimicrobials. It is unlikely that there is a significant difference in bacterial coverage. However, because of our low rate of positive cultures we are unable to examine whether there was significant antimicrobial resistance. Previous studies have demonstrated that macrolides have significant anti-inflammatory effects [30-33] and this might be the explanation for our results. For example, there is a substantial body of research that demonstrates that elevated cytokine levels are associated with septic shock or acute respiratory distress syndrome [34-37]. In addition many of the negative prognostic factors for patients with community-acquired pneumonia, such as fever, leukopenia, hypoalbuminemia, and hypotension, are mediated by individual cytokines [36,38-40]. It is therefore plausible that this anti-inflammatory effect is most pronounced in this population, who would have a higher level of serum cytokines than patients with less severe cases of pneumonia. We therefore find it unlikely that the effect on mortality is due to the specific combination of the β-lactam plus fluoroquinolone. Rather, our hypothesis is that regimes that contain macrolides have significant protective effects for patients with pneumonia.

Our study has several limitations that should be acknowledged. First, it was a retrospective cohort study, and inherent problems related to this design include ascertainment bias. However, we do not feel that this study has problems with ascertainment bias because our method used discharge diagnosis ICD-9 codes to identify patients. Second, our sample was predominantly male owing to the inclusion of a Department of Veterans Affairs hospital and it is possible, but unlikely, that females might have a different responsiveness to antibiotics from that of males. Third, we were unable to collect information on cause of death or re-hospitalizations on this cohort, so we were unable to examine these outcomes. Fourth, we collected information on the use of mechanical ventilation within the first 24 hours only, so we may have missed patients who required mechanical ventilation after that. Finally, as in any non-experimental study, we are unable to state conclusively that the empiric use of a β-lactam with a fluoroquinolone is the cause of increased mortality in this cohort. However, we have no reason to believe that β-lactam alone or with fluoroquinolones is more likely to be given to patients who present with more severe illness. In addition, the use of the propensity score provided a way to control for these differences in the analysis by defining patients comparable to those with the same score.

## Conclusion

This study demonstrates an association for patients hospitalized with severe community-acquired pneumonia between the empiric use of a β-lactam with a fluoroquinolone and increased 30-day mortality. These results call into question the recommendation for the use of a β-lactam and fluoroquinolone in patients with severe community-acquired pneumonia. Further research is needed to examine this combination of antibiotics and to determine the best antibiotic combinations for patients hospitalized with severe community-acquired pneumonia.

## Key messages

• 	There has been little research comparing the different empiric guideline-concordant antimicrobial therapies for patients with severe community-acquired pneumonia.

• 	The empiric use of a β-lactam plus fluoroquinolone was associated with higher 30-day mortality than with other guideline-concordant regimes for patients hospitalized with severe pneumonia.

• 	Further research is needed to determine the ideal empiric antibiotic regimes for patients with severe pneumonia.

## Competing interests

None of the authors, except AA, have any conflicts of interest to disclose regarding this paper. AA is currently a consultant with Pfizer, Ortho-McNeil, and Bayer Pharma.

## Authors' contributions

EMM originated and coordinated the study, obtained funding, contributed to the analysis of the data, and contributed to preparation of the paper. MIR, AA, and JP contributed to the design of the study, the analysis of the data, and preparation of the paper. All authors read and approved the final manuscript.

## Figures and Tables

**Table 1:** Subject demographic and clinical characteristics by 30-day mortality

Variable	30-day mortality		*p*
		
	Alive (*n *= 138)	Dead (*n *= 34)	
Age (years)	63.9 ± 16	61.8 ± 14	0.2
Men	123 (90)	27 (79)	0.1
Nursing home resident	14 (10)	4 (11)	0.7
Emergency department admission	126 (91)	30 (88)	0.6
Admitted to intensive care =24 hours	82 (59)	24 (71)	0.6
Mechanical ventilation	52 (37)	3 (8)	<0.001
Pre-existing comorbid conditions			
Congestive heart failure	36 (26)	5 (15)	0.2
Chronic pulmonary disease	45 (33)	8 (24)	0.3
History of stroke	30 (22)	4 (12)	0.2
Chronic liver disease	13 (9)	6 (18)	0.17
History of malignancy	13 (9)	11 (32)	0.001
Renal insufficiency	4 (3)	0	0.3
History, physical, laboratory, and radiographic data			
Altered mental status	33 (24)	9 (26)	0.8
Respiratory rate >30 per minute	29 (21)	8 (24)	0.7
Systolic blood pressure <90 mmHg	6 (4)	1 (3)	0.7
Heart rate >125 per minute	29 (21)	14 (41)	0.015
Temperature <95°C or >104°C	7 (5)	2 (6)	0.8
Arterial pH <7.35	25 (18)	11 (32)	0.07
Arterial oxygenation <90%	52 (38)	16 (47)	0.3
Hematocrit <30%	21 (15)	2 (6)	0.15
Blood urea nitrogen >30 mg/dl	58 (42)	15 (44)	0.8
Serum glucose >250 mg/dl	21 (15)	3 (8)	0.3
Serum sodium <130 meq/l	30 (22)	8 (24)	0.8
Pleural effusion	48 (35)	17 (50)	0.1
Multilobar infiltrates	64 (47)	18 (52)	0.5
Pneumonia severity index			
Class I to III	34 (25)	6 (18)	
Class IV	40 (29)	8 (24)	
Class V	64 (46)	20 (58)	0.4
Processes of care			
Initial antibiotics within 4 hours	46 (30)	10 (29)	0.7
Blood cultures prior to antibiotics	110 (79)	29 (85)	0.45
Oxygenation assessed =24 hours	120 (87)	30 (88)	0.8

Data are presented as number (%) or mean ± SD.

**Table 2:** Subject demographic and clinical characteristics by use of a β-lactam plus fluoroquinolone versus non-use

Variable	β-lactam plus fluoroquinolone		*p*
		
	Use (*n *= 50)	Non-use (*n *= 122)	
Age (years)	62.9 ± 14	63.8 +/- 16	0.4
Men	44 (88)	106 (87)	0.9
Nursing home resident	10 (20)	8 (7)	0.009
Emergency department admission	46 (92)	110 (90)	0.7
Admitted to intensive care =24 hours	37 (74)	69 (57)	0.03
Mechanical ventilation	21 (38)	34 (28)	0.08
Pre-existing comorbid conditions			
Congestive heart failure	10 (20)	31 (25)	0.04
Chronic pulmonary disease	18 (36)	35 (28)	0.3
History of stroke	12 (24)	22 (18)	0.4
Chronic liver disease	6 (12)	13 (10)	0.8
History of malignancy	6 (12)	18 (15)	0.6
Renal insufficiency	11 (22)	25 (20)	0.8
History, physical, laboratory, and radiographic data			
Altered mental status	13 (26)	29 (24)	0.8
Respiratory rate >30 per minute	12 (24)	25 (20)	0.6
Systolic blood pressure <90 mmHg	1 (2)	6 (5)	0.4
Heart rate >125 per minute	17 (34)	26 (21)	0.08
Temperature <95°C or >104°C	5 (10)	4 (3)	0.07
Arterial pH <7.35	14 (28)	22 (18)	0.15
Arterial oxygenation <90%	15 (30)	53 (43)	0.1
Hematocrit <30%	3 (6)	20 (16)	0.07
Blood urea nitrogen >30 mg/dl	24 (48)	49 (40)	0.3
Serum glucose >250 mg/dl	6 (12)	18 (15)	0.6
Serum sodium <130 meq/l	19 (20)	19 (24)	0.6
Pleural effusion	20 (40)	45 (37)	0.7
Multi-lobar infiltrates	24 (48)	58 (48)	0.9
Pneumonia severity index			
Class I to III	13 (26)	27 (22)	
Class IV	14 (28)	34 (28)	
Class V	23 (46)	61 (50)	0.8
Processes of care			
Initial antibiotics within 4 hours	13 (26)	43 (35)	0.4
Blood cultures prior to antibiotics	41 (82)	98 (80)	0.8
Oxygenation assessed =24 hours	42 (84)	108 (89)	0.4

Data are presented as number (%) or mean ± SD.

**Table 3:** Etiologies of severe community-acquired pneumonia

Microorganisms	Number (*n *= 41)
*Streptococcus pneumoniae*	15
*Staphylococcus aureus*	10
*Pseudomonas aeruginosa*	2
*Haemophilus influenzae*	7
Enterobacteriacea^a^	3
Miscellaneous^b^	2
Other Gram-positive cocci^c^	2

^a^Including *Escherichia coli*, *Klebsiella pneumoniae*, *Klebsiella oxytoca*, and *Proteus mirabilis*; ^b^*Acinetobacter *species, *Aspergillus *species, and *Haemophilus parainfluenzae*; ^c^including *Enterococcus *species and *Streptococcus *species.

**Table 4:** Results of multivariable logistic regression model

Variable	β coefficient	SEM	95% CI	*p*
Propensity score	-0.423	0.183	-0.78 to -0.65	0.02
Use of β-lactam plus fluoroquinolone	0.9985	0.413	0.19 to 1.81	0.016
Intercept	-1.15	0.323	-1.79 to -0.519	<0.001

CI, confidence interval.
